# Enhancing Caries Preventive Effects of Nanomaterials with Phototherapy: A Scoping Review

**DOI:** 10.3390/jfb16090308

**Published:** 2025-08-26

**Authors:** Veena Wenqing Xu, Iris Xiaoxue Yin, John Yun Niu, Chun-Hung Chu

**Affiliations:** Faculty of Dentistry, University of Hong Kong, Hong Kong 999077, China; u3008489@connect.hku.hk (V.W.X.); irisxyin@hku.hk (I.X.Y.); niuyun@hku.hk (J.Y.N.)

**Keywords:** nanomaterials, caries, prevention, antibacterial, phototherapy

## Abstract

The objective of this study was to provide a comprehensive review of the types, properties, and potential applications of nanomaterials in phototherapy for caries prevention. This scoping review follows the Preferred Reporting Items for Systematic reviews and Meta-Analyses extension for Scoping Review (PRISMA-ScR). Two researchers independently searched English-language publications in Pubmed, Embase, and Web of Science on 25 February 2025. Publications that reported nanomaterials in phototherapy for caries prevention are included. They screened 229 publications and included 38 publications. These 38 publications were categorised into three groups: nanomaterials in photodynamic therapy (25/38, 66%), nanomaterials in photothermal therapy (9/38, 24%), and nanomaterials in combined photothermal and photodynamic therapy (4/38, 10%). Nanomaterials in photodynamic therapy generate reactive oxygen species under light, causing oxidative damage that kills microbes. In photothermal therapy, nanomaterials convert light energy into heat, inducing protein denaturation and membrane rupture, which eliminate microbes. These nanomaterials were incorporated into dental materials like adhesives and topical anti-caries agents. Among the 38 publications, 29 were laboratory studies, 8 were animal studies, and 1 was a human trial. Studies showed that some nanomaterials inhibit cariogenic microbes under light. However, most of the studies were laboratory or animal studies. More human trials are essential to translate their use into clinical care. This review underscores the potential of nanomaterials in phototherapy—leveraging photodynamic and photothermal mechanisms to eliminate caries-causing microbes—as a promising, minimally invasive strategy for caries prevention.

## 1. Introduction

Dental caries is a widespread chronic oral disease affecting over 2.4 billion people globally [1]. It ranks as the fourth most costly disease to treat, adding significantly to the human health burden [2]. The pathogenesis of dental caries is multifactorial, involving host factors, bacteria, substrate, and time, with cariogenic biofilms playing a central role [3]. These biofilms enable microbes to metabolise fermentable carbohydrates into organic acids, which can dissolve the mineralised hard tissues of enamel and dentine [3]. Continuous demineralisation leads to the destruction of the tooth structure, culminating in dental caries [4]. Thus, effective management strategies are essential to prevent dental caries.

Current caries management strategies focus on inhibiting bacterial activity and biofilm growth to reduce acid production and demineralisation [5]. Treatments include mechanical removal of carious tissue, fluoride therapy, and antimicrobial agents [6]. However, these methods are limited by invasiveness, incomplete bacterial eradication [7], risk of resistance [8], and side effects such as fluorosis [9]. Given these challenges, innovative, non-invasive approaches that can effectively target biofilms without promoting resistance are urgently needed.

Phototherapy, a non-invasive technique utilising light to treat various conditions, has gained attention in medical fields such as dermatology and oncology [10,11]. In dentistry, two primary types of phototherapies, photodynamic therapy and photothermal therapy, have shown promise in targeting oral biofilms and pathogenic microbes [12]. In photodynamic therapy, photosensitising agents produce reactive oxygen species when exposed to a specific wavelength of light [13]. These reactive oxygen species cause oxidative damage at the molecular level, such as lipid peroxidation, deoxyribonucleic acid strand breaks, and protein oxidation, which disrupts microbial cell function and integrity [14]. On the other hand, photothermal agents convert light energy into heat, resulting in increased local temperatures that cause protein denaturation by altering protein structure and function, as well as rupturing cell membranes, ultimately leading to microbial cell death [15]. Compared to traditional methods, phototherapy offers precise, non-invasive treatment with a lower risk of inducing bacterial resistance [16,17]. Recent studies have shown significant promise for phototherapy in treating oral diseases and combating microbial infections [18,19].

**Figure 1:**
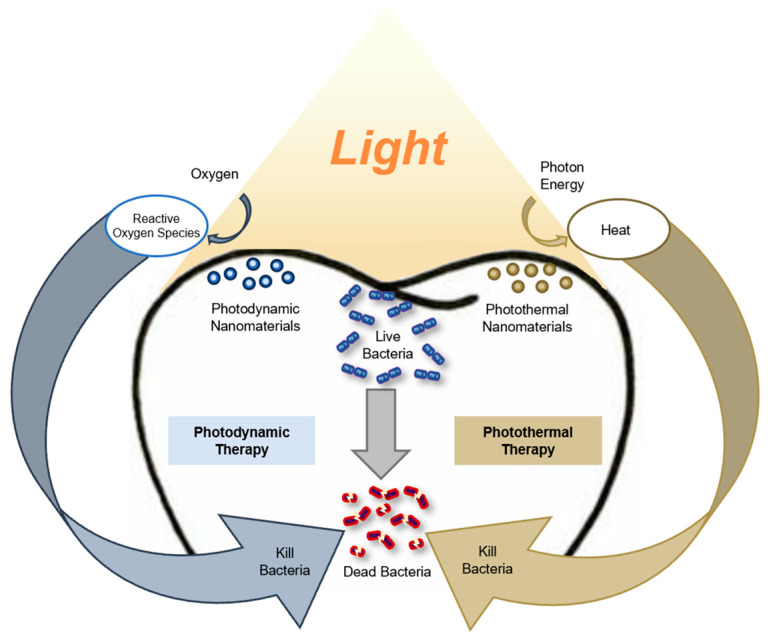
Mode of phototherapy for preventing dental caries.

Advancements in nanotechnology have further enhanced the potential of phototherapy for caries prevention. Nanomaterials, owing to their high surface area, tuneable properties, and ability to facilitate targeted delivery, can improve the efficacy and selectivity of phototherapeutic agents [20,21]. These materials can be engineered for precise drug release in response to specific stimuli like pH, temperature, or light [22]. Furthermore, nanomaterials can be designed to protect therapeutic agents from degradation or to enhance the solubility of hydrophobic drugs, thereby improving their stability and bioavailability [23]. In general phototherapy, a variety of nanomaterials have been developed and applied. For example, gold nanoparticles have been widely used as photothermal agents for tumour ablation in oncology [24]. In addition, graphene oxide nanosheets have been employed to enhance phototherapy for cancer treatment [25]. In dentistry, the integration of nanomaterials with phototherapy has shown great promise for preventing dental caries. Nanomaterials not only amplify photophysical and photothermal responses, enabling more effective disruption of cariogenic biofilms, but also serve as efficient carriers that facilitate deeper penetration into biofilms and sustained antibacterial effects [26]. Phototherapy activates these nanomaterials at the target site, enhancing their antimicrobial effect to achieves more potent biofilm eradication [27]. Depending on the phototherapeutic modality, various nanomaterials have been developed for caries prevention; for example, curcumin nanoparticles and titanium dioxide nanoparticles can generate reactive oxygen species for photodynamic therapy [28,29], while gold nanoparticles and black phosphorus nanosheets are used to convert light into heat for photothermal therapy [30,31]. Some advanced nanomaterials, such as graphene oxide nanosheets, are engineered to possess both photodynamic and photothermal properties, enabling combined therapeutic effects [32]. Figure 1 illustrates various phototherapy modes for preventing dental caries.

Despite the promising advances, there is currently no comprehensive review summarising the applications of nanomaterials in phototherapy for dental caries prevention. The objective of this study is to systematically examine the types, properties, and therapeutic applications of nanomaterials in phototherapy for caries prevention. By consolidating current knowledge, this work seeks to guide the future development of novel, non-invasive strategies for effective caries prevention.

## 2. Methods

### 2.1. Search Strategy

This scoping review follows the Preferred Reporting Items for Systematic reviews and Meta-Analyses extension for Scoping Review (PRISMA-ScR) [33]. The PRISMA-ScR checklist is provided in the Supplementary Material. The protocol was published on the Open Science Framework on 13 June 2025 (https://doi.org/10.17605/OSF.IO/YEKSZ). Two independent investigators conducted a literature search across three widely used databases, namely PubMed, Embase, and Web of Science, to identify relevant studies. The search terms included (photothermal OR photodynamic OR phototherapy) AND (caries OR dental decay OR tooth decay OR carious lesion OR white spot) AND (nano OR nanomaterials OR nanoparticles OR NPs OR nanocomposites OR nanocomplexes). The search was restricted to studies published in English, with no limitations on the publication date. The final search was completed on 25 February 2025.

### 2.2. Study Selection and Data Extraction

**Figure 2:**
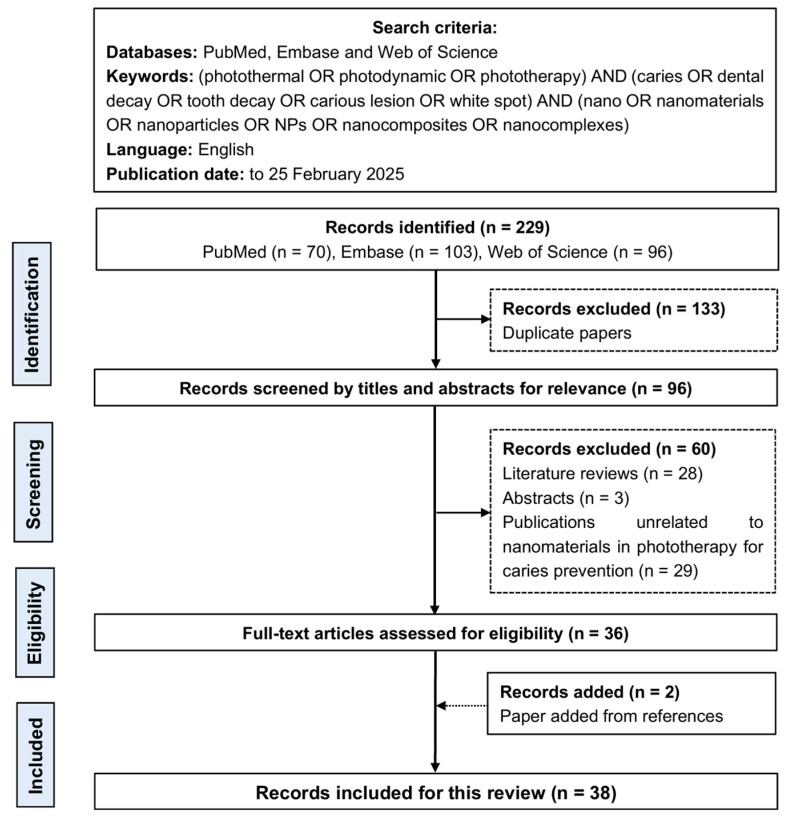
Flow chart of the literature search, based on the PRISMA 2020 flow diagram.

This scoping review included original studies of nanomaterials in phototherapy for caries prevention (Figure 2).

Two researchers independently reviewed and removed duplicate entries to compile a list of relevant publications. They then screened the titles and abstracts to identify original studies focusing on nanomaterials in phototherapy for caries prevention. Publications such as literature reviews, abstracts, and studies not related to the use of nanomaterials in phototherapy for caries prevention were excluded. The researchers retrieved the full texts of the remaining articles for a more detailed examination. They specifically selected studies focusing on nanomaterials in phototherapy for preventing caries. Additionally, they manually checked the reference lists of these selected studies to find any other eligible publications. To finalise the list, they consulted with a third researcher to reach a consensus on which studies to include in the review. The remaining original studies focusing on nanomaterials in phototherapy for preventing caries were included in this review. The researchers documented information on each publication, noting details such as the nanomaterials used, study design, anti-caries properties investigated, the type of phototherapy applied, the light wavelength and exposure time, and potential applications.

### 2.3. Assessment of the Risk of Bias

Two investigators independently evaluated the risk of bias in each study. Table 1 presents the methods used to assess the risk of bias in the included in vitro, animal, and human studies.

## 3. Results

The initial literature search identified 229 potential publications: 70 from PubMed, 103 from Embase, and 96 from Web of Science. After removing 133 duplicate records, 96 unique publications remained. The researchers reviewed the titles and abstracts and excluded 60 publications that were either literature reviews, abstracts, or unrelated to the use of nanomaterials in phototherapy for caries prevention. An additional two eligible studies were identified by screening the reference lists of selected publications. Ultimately, a total of 38 publications were included in this review.

The majority of the included studies (29 out of 38 studies, 76%) were in vitro experiments, followed by animal studies (8 out of 38 studies, 21%), with only 1 human study (1 out of 38 studies, 3%) identified. Regarding the application of nanomaterials, 16 studies (42%) involved their incorporation into topical agents, 6 (16%) into dental adhesives, 4 (11%) into restorative fillers, and 1 study each (3%) investigated oral capsules, buccal films, pulp capping agents, orthodontic brackets, or gargle. In eight studies (21%), the specific application was not specified. With respect to the type of therapy, most studies investigated nanomaterials for photodynamic therapy (25 out of 38 studies, 66%), followed by photothermal therapy (9 out of 38 studies, 24%) and combined photodynamic and photothermal therapy (4 out of 38 studies, 10%).

The risk of bias in these 38 publications was evaluated. Table 2 shows the risk of bias of the 29 in vitro studies. Of these, 28 studies were rated as having some concerns, while only 1 study was assessed as having a low risk of bias. Table 3 shows the risk of bias of the eight animal studies. All animal studies were rated as low risk. Table 4 shows the risk of bias of the only human study, which was rated as low risk.

### 3.1. Nanomaterials in Photodynamic Therapy

When irradiated with light of an appropriate wavelength, nanomaterial-bound or -encapsulated photosensitisers are excited to a higher energy state [70]. Subsequently, they transfer energy to molecular oxygen, producing singlet oxygen and other reactive oxygen species [71]. These reactive species induce oxidative damage to bacterial structures, such as cell walls and membranes [72]. The incorporation of nanomaterials enhances this effect by increasing the uptake of photosensitisers by bacteria, improving penetration into biofilms, and protecting the photosensitisers from premature degradation [73]. Recent advances in nanotechnology have enabled the development of a wide range of nanomaterial-mediated photodynamic therapy strategies for the prevention of dental caries [74]. These findings are consistent with previous reviews, which also highlight the role of nanomaterials in photodynamic therapy [75,76]. Table 5 summarises 25 publications on the use of nanomaterials in photodynamic therapy for caries prevention, including one human study, four animal studies, and the remainder being in vitro investigations.

#### 3.1.1. Natural Photodynamic Nanomaterials

Natural compound-based nanomaterials, particularly those incorporating curcumin, emodin, or quercetin, have emerged as promising biocompatible alternatives for photodynamic therapy for caries prevention [77]. The two key limitations of these natural compounds are poor solubility and low bioavailability. Nanotechnology strategies can solve the challenges by encapsulation or formulation into nanomicelles or nanoparticles [78].

Curcumin, a hydrophobic polyphenol extracted from turmeric, is well-known for its anti-inflammatory and antimicrobial properties [79]. Its potential in photodynamic therapy arises from its capacity to generate reactive oxygen species upon activation with blue light [80]. Previous studies showed that encapsulation within nanomicelles or nanoparticles significantly improves curcumin’s solubility, cellular uptake, and photodynamic effect [81,82].

An in vitro study demonstrated that curcumin nanomicelles, when activated by a 450 nm dental light-emitting diode for 120 s, resulted in a substantial reduction of 99.5% in the viability of *Lactobacillus casei* (*L. casei*) [59]. The nanomicellar formulation outperformed free curcumin, underscoring the advantages of nanoscale delivery systems in enhancing photodynamic therapy outcomes. Another study evaluated buccal films loaded with curcumin nanoparticles, which provided sustained local release and effectively inactivated 99.9% of *Streptococcus mutans* (*S. mutans*) under blue light irradiation for 12 min [52]. Two studies investigated topical gel formulations of curcumin nanoparticles, demonstrating efficacy against both *S. mutans* and *Lactobacillus acidophilus* (*L. acidophilus*), thus indicating broad-spectrum antimicrobial potential [38,56]. Pourhajibagher et al. developed a curcumin-containing pulp-capping agent which, when combined with blue light photodynamic therapy, conferred durable antibacterial effects lasting up to 60 days, supporting its potential application in direct pulp capping for deep carious lesions [58]. In addition, a clinical study in people receiving orthodontic care utilised oral curcumin nanomicelle capsules, resulting in a significant reduction in *S. mutans* counts and remineralisation of white spot lesions, demonstrating both antibacterial and remineralising efficacy [69]. Curcumin nanoparticles have also been incorporated into dental adhesives, maintaining bond strength while imparting anti-biofilm properties [29,62].

Emodin, a naturally occurring anthraquinone commonly isolated from the roots and rhizomes of several medicinal plants such as Rheum palmatum, has also exhibited potent antimicrobial effects in photodynamic therapy when formulated as nanoparticles [83]. A study reported that emodin–chitosan nanoparticles could be photo-activated to reduce 99.9% of *S. mutans* biofilms on enamel surfaces [40].

Quercetin, a natural flavonoid isolated from many fruits and vegetables such as onions and apples, has been similarly formulated as nanoparticles to enhance its photodynamic therapy effect [84]. A study showed that quercetin nanoparticles effectively inhibit bacterial growth and reduced the number of viable *S. mutans* by 99% by downregulating virulence gene expression under blue light activation [49]. In another study, quercetin nanoparticles were combined with propolis quantum dots, with nisin as a topical agent for the prevention of white spot lesions, providing a natural and biocompatible approach to caries prevention [53].

#### 3.1.2. Synthetic Photodynamic Nanomaterials

Synthetic photodynamic nanomaterials are engineered materials widely employed in photodynamic therapy due to their high yields of reactive oxygen species and strong photo-absorption ability [85]. Encapsulation or conjugation with nanoparticles enhances the solubility, stability, and targeted delivery of photosensitisers, thereby facilitating more efficient penetration and retention within oral biofilms [86]. Consequently, these materials offer reliable and potent antimicrobial effects, rendering them highly suitable for incorporation into topical agents, dental adhesives, and restorative materials for the prevention and treatment of dental caries.

Toluidine blue ortho is a photosensitiser. When irradiated with red light, toluidine blue ortho nanoparticles exhibit strong antibacterial activity against *S. mutans* and more complex saliva-derived biofilms [48]. Notably, combining propolis with toluidine blue ortho nanoparticles results in greater inhibition of bacterial growth compared to propolis alone [50]. Another study developed a multifunctional nanoplatform integrating toluidine blue ortho with superparamagnetic iron oxide nanoparticles; under an external magnetic field, this system facilitated deeper penetration into biofilms, significantly enhancing the photodynamic disinfection effect relative to toluidine blue ortho alone [42].

Methylene blue is also a photosensitiser. Methylene blue nanoparticles, particularly those combined with titanium oxide and chitosan, provide both photodynamic bacterial inhibition and reinforcement of dentine adhesive properties [87]. Upon light activation, methylene blue nanoparticles generate reactive oxygen species, such as singlet oxygen and free radicals, which cause oxidative damage to bacterial cell membranes, proteins, and DNA, ultimately leading to bacteria death [88]. Research has demonstrated effective disinfection of caries-affected dentine while maintaining bond strength, indicating the potential of these materials for clinical adhesive systems [37]. Another study showed that methylene blue nanoparticles showed high antibacterial effects, effectively killed 99% of *Lactobacillus acidophilus* (*L. acidophilus*), and they can be applied as a topical agent [44].

Chlorin e6 is a semi-synthetic derivative of chlorophyll [89]. It is recognised for its potent photosensitising properties and strong absorption in the red region of the spectrum. Its high reactive oxygen species yield makes it particularly effective for photodynamic therapy [90]. A study showed that chlorin e6 combined with copper and zeolitic imidazolate framework-8 formed an intelligent acid-responsive photosensitive material that reduce biofilm formation by 84.7% in vitro [54]. Chlorin e6 nanoparticles have also been shown to significantly increase penetration depth and retention within biofilms compared to the free compound [68]. Additionally, hydroxyapatite–chlorin e6 composites have been developed to prevent demineralisation and inhibit *S. mutans* [46]. A novel gargle formulation containing chlorin e6, combined with a saliva-acquired peptide to enhance adherence to dental surfaces, demonstrated prolonged antibacterial effects [65].

Zinc oxide and titanium dioxide nanoparticles serve both as antimicrobial agents in the absence of light and as photosensitisers [91,92]. Studies have shown that zinc oxide nanoparticles can be incorporated into adhesives and restorative fillers, significantly reducing *S. mutans* viability under blue light and contributing to anti-demineralisation effects [39,43]. Furthermore, titanium dioxide nanoparticles provide photocatalytic inactivation of biofilms when used in topical agents and adhesives [28,60]. These results agree with prior research indicating that titanium dioxide nanoparticles offer significant advantages in antimicrobial effects for photodynamic oral treatment [93,94].

### 3.2. Nanomaterials in Photothermal Therapy

Photothermal therapy is an emerging antimicrobial strategy for caries prevention. Upon irradiation with near-infrared or visible light, photothermal nanomaterials efficiently convert photon energy into heat, elevating local temperatures to bactericidal levels, often exceeding 50 °C [95]. This localised hyperthermia disrupts bacterial membranes, denatures proteins, and can promote biofilm detachment [96]. Importantly, the targeted heating minimises collateral tissue damage. This allows repeated and non-invasive application. Photothermal therapy is highly effective at breaking down mature antibiotic-resistant biofilms [97]. Table 6 summarises the nine publications on nanomaterials for photothermal therapy in caries prevention, comprising three animal and six laboratory studies.

#### 3.2.1. Metal-Based Photothermal Nanomaterials

Metal nanoparticles, notably gold, bismuth, and iron (III) ferrocyanide (prussian blue), are at the forefront of dental photothermal therapy research owing to their excellent photothermal conversion effect, biocompatibility, and functionalisation potential [98,99].

Gold nanoparticles display strong light absorption capability in the visible region (about 380 nanometers to 740 nanometers) and near-infrared region (about 750 nanometers to 2500 nanometers) due to surface plasmon resonance, a collective oscillation phenomenon at their surface [100]. When irradiated with near-infrared light, they rapidly generate localised heat, enabling the destruction of biofilms while minimising damage to the surrounding healthy tissues [101]. Additionally, their tuneable size and surface chemistry allow for functionalisation with targeting ligands or therapeutic agents, making them a highly promising platform for precise and effective photothermal therapy in dentistry and other biomedical applications [102]. For example, dental resins loaded with gold nanoshells under light resulted in more than 99% reductions in *S. mutans* [57]. Further, an animal (rat) study showed that gold-in-gold cage nanoparticles have potent photothermal ablation activity against *S. mutans* and *Staphylococcus aureus* (*S. aureus*) [30]. Their antibacterial efficacy is more than 100 times more than chlorhexidine, a conventional antimicrobial agent.

Bismuth nanoparticles have also emerged as promising nanomaterials for biomedical applications due to their unique physicochemical properties, such as facile functionalisation, strong X-ray attenuation, and excellent biocompatibility [103]. They exhibit high photothermal conversion efficiency, making them attractive for photothermal therapy [104]. Bismuth quantum dot/polydimethylsiloxane composites with photothermal antibacterial property were added as restorative fillers into restorative materials [55]. The restorative materials could effectively eradicate 99% of *S. mutans* upon light exposure.

Iron (III) ferrocyanide (Prussian blue) is an emerging photothermal agent that also provides antibacterial effects through ion release [105]. Silver-doped Prussian blue hydrogels under light rapidly eradicated *S. mutans*, *Streptococcus sobrinus* (*S. sobrinus*), and *Streptococcus sanguinis* (*S. sanguinis*) [67].

Two-dimensional transition metal carbides or nitrides (MXenes) possess unique photothermal and mechanical properties [106]. A study showed that titanium aluminium carbide nanosheets, one type of MXene, can eradicate more than 90% of *S. mutans* biofilms in vitro when incorporated into dental resin and exposed to natural light [61]. This finding underscores their potential application as antibacterial and mechanically reinforcing fillers in restorative dental materials.

#### 3.2.2. Carbon-Based Photothermal Nanomaterials

Carbon-based nanomaterials, such as graphene oxide and carbon nanoparticles, exhibit excellent photothermal properties owing to their broad-spectrum light absorption and high surface area [107]. Researchers developed graphene oxide nanosheets that combine photothermal, antibacterial, and mechanical reinforcement properties [51]. The graphene oxide nanosheets were able to effectively disrupt *S. mutans* biofilms and caused 98% viability loss upon near-infrared irradiation for 5 min.

Organic nanomaterials, particularly polymeric nanomaterials such as polydopamine nanoparticles, are designed for biocompatibility and flexibility [108]. Researchers developed removable photothermal antibacterial “warm paste” nanoagents by modifying silver nanoparticles with polydopamine [45]. Under near-infrared irradiation, these nanoparticles provide effective antibacterial action through photothermal therapy, functioning similarly to a traditional “warm paste”. Another study described a nanohybrid comprising stabilised amorphous calcium phosphate loaded onto polydopamine nanoparticles [47]. This system achieves both biomimetic remineralisation and biocompatible near-infrared photothermal therapy. The results demonstrated that the nanohybrid was highly bactericidal and promoted the remineralisation of demineralised enamel.

#### 3.2.3. Phosphorus-Based Nanomaterials

Black phosphorus nanosheets, noted for their strong light absorption capability and biocompatibility, have been integrated into mussel-inspired hydrogels to provide wet-tissue adhesion and photothermal antibacterial effects [109]. Notably, black phosphorus can release phosphate ions, which are essential for biomineralization processes in hard tissues such as teeth and bone [110]. A study showed that black phosphorus–hydrogel composites prevent caries by quickly eradicating 99% of *S. mutans* and *S. sanguinis* [31]. The composites also promote enamel remineralisation in vitro and in rats.

### 3.3. Nanomaterials in Combined Photothermal and Photodynamic Therapy

Dual-modal nanomaterials provide synergistic antimicrobial effects by combining rapid, heat-induced bacterial inactivation (photothermal therapy) with reactive oxygen species-mediated damage (photodynamic therapy) [111]. This approach enhances antimicrobial effects, facilitates deeper biofilm penetration, and reduces the likelihood of bacterial resistance [112]. Table 7 summarises four publications investigating dual-modal nanomaterials for caries prevention, comprising two animal studies and two in vitro studies.

#### 3.3.1. Metal-Based Dual-Modal Nanomaterials

Zinc phthalocyanine, a synthetic macrocyclic compound from the phthalocyanine family, is notable for its strong absorption of red and near-infrared light. Its strong absorption enables efficient activation and deep tissue penetration during phototherapy [113]. One study developed a zinc phthalocyanine-based supramolecular nanoformulation capable of switching between photothermal and photodynamic effects according to the local microenvironment [63]. The nanoformulation eliminated 99% of the bacteria in the rat model.

#### 3.3.2. Carbon-Based Dual-Modal Nanomaterials

Carbon-based materials, especially graphene oxide and reduced graphene oxide, are well-suited for dual-modal therapy owing to their high surface area and tuneable optical properties [114]. In one study, graphene oxide nanoparticles were incorporated into orthodontic adhesives [41]. The dual-modal therapy resulted in 90% reductions in *S. mutans* biofilms while also improving shear bond strength. Reduced graphene oxide nanoparticles were used on elastomeric orthodontic ligatures to kill *S. mutans* by both photothermal and photodynamic effects [32]. This dual-modal coating shows promise for preventing biofilm formation and the associated enamel demineralisation around orthodontic appliances. These studies are in line with previous studies, which have reported that graphene oxide nanoparticles can produce synergistic antimicrobial effects [12,94].

Organic, particularly polymeric, nanoplatforms can be engineered to deliver both photothermal and photodynamic effects [115]. Researchers designed a near-infrared-activated polymeric nanosystem with self-modulating bacterial targeting properties [64]. This system can be applied as a topical gel or hydrogel and achieved effective eradication of biofilms in vitro and in rat models. Such dual-modal approaches are particularly advantageous for the treatment of persistent, mixed-species biofilms commonly found in caries lesions.

## 4. Discussion

The search strategy of this scoping review combined free-text terms and Medical Subject Headings (MeSH) vocabulary, such as “phototherapy,” “dental caries,” “nanomaterials,” and “nanoparticles,” to maximise the retrieval of relevant studies. This dual approach was critical, as not all studies are consistently indexed with MeSH terms in scientific databases. To further enhance this review’s comprehensiveness and mitigate potential gaps in database indexing, the researchers manually screened the reference lists of all included studies to identify additional eligible publications.

This review was conducted using PubMed, Embase, and Web of Science as the primary sources of studies. These databases were selected for their broad and comprehensive coverage of peer-reviewed scientific research, providing a strong foundation for this review [116]. However, some relevant studies may have been excluded if they were published in journals not indexed by these databases. In addition, as these platforms predominantly index peer-reviewed journal articles, important insights from books or reports produced by organisations may have been overlooked [117]. Additionally, this study only included publications written in English. Although English is widely used in academic publishing and many influential studies appear in English-language journals, this restriction could lead to bias [118]. Research from various regions and cultural backgrounds may offer distinct insights and perspectives. By excluding non-English sources, valuable result from these studies might be missed [119].

The review found that only a small number of studies exhibited a low risk of bias. Although biocompatibility is a key consideration in the evaluation of biomaterials, over half of the included studies did not report on the biocompatibility of the nanomaterials investigated. Therefore, there is a clear need for more high-quality research that examines the use of nanomaterials in phototherapy for caries prevention. Furthermore, although the literature reported plenty of nanomaterials in phototherapy for caries prevention, most publications are laboratory studies. Well-designed human studies are essential to confirm their clinical effectiveness and safety. Similar calls for more robust clinical data have been made in previous reviews about nanomaterials in dentistry, highlighting this as an ongoing gap in the field [120,121].

## 5. Conclusions

The literature reported that nanomaterial-enabled phototherapies represent promising strategies for the prevention of dental caries. In photodynamic therapy, both natural and synthetic nanomaterials facilitate targeted, reactive oxygen species-mediated antimicrobial effects. Most studies focus on metal-based nanoparticles combined with photodynamic therapy, demonstrating antibacterial effects in vitro. Photothermal therapy, employing metal-based, carbon-based, and phosphorus-based nanomaterials, enables rapid and localised biofilm eradication, particularly in challenging or high-risk sites. Combined photothermal and photodynamic therapy, which is supported by metal-based and carbon-based nanomaterials, offers synergistic and comprehensive antimicrobial action suitable for complex clinical scenarios. These technologies are being developed in a variety of dental delivery formats, including adhesives, restorative fillers, topical gels, coatings, and oral capsules, demonstrating considerable versatility and clinical potential. Nevertheless, most of the current evidence is preclinical, and there is a lack of standardised protocols as well as limited long-term safety data. More human trials using standardised methodologies and evaluating long-term safety are essential to facilitate the integration of these promising nanomaterial-based phototherapies into routine dental practice.

## Figures and Tables

**Table 1:** Risk of bias assessment methods for the included in vitro, animal, and human studies.

Study Type	Assessment Tool [Ref.]	Criteria	Scoring System	Risk of Bias Classification
In vitro studies	Quality Assessment Tool for In Vitro Studies (QUIN Tool) [34]	(1) Clearly stated aims/objectives; (2) sample size calculation; (3) sampling technique; (4) details of comparison groups; (5) detailed methodology; (6) operator details; (7) randomisation; (8) measurement of outcomes; (9) outcome assessor details; (10) blinding; (11) statistical analysis; (12) presentation of results	Low risk (2 points); some concerns (1 point); high risk (0 points)	>70%: low risk; 50–70%: some concerns; <50%: high risk
Animal studies	Systematic Review Centre for Laboratory Animal Experimentation (SYRCLE’s) Risk of Bias Tool [35]	(1) Allocation sequence; (2) baseline similarity; (3) allocation concealment; (4) random housing of animals; (5) caregiver/investigator blinding; (6) random outcome assessment; (7) outcome assessor blinding; (8) incomplete data addressed; (9) free of selective reporting; (10) free of other biases		
Human studies	Cochrane Risk of Bias Tool for Randomised Trials Version 2 (RoB 2) [36]	(1) Randomization process bias; (2) intervention deviation bias; (3) missing outcome data; (4) outcome measurement bias; (5) reporting selection bias		

**Table 2:** Risk of bias of the included in vitro studies *.

First Author, Year [Ref.]	Items of Assessment												Score (%)	Risk of Bias
	Clearly Stated Aims/Objectives	Sample Size Calculation	Sampling Technique	Details of Comparison Group	Detailed Methodology	Operator Details	Randomisation	Measurement of Outcome	Outcome Assessor Details	Blinding	Statistical Analysis	Presentation of Results		
Alanazi, 2024 [37]	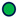	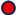	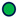	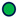	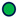	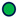	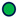	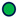	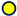	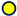	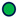	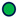	83	Low
Afrasiabi, 2024 [38]	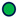	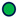	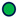	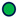	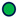	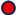	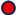	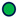	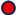	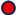	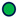	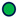	67	Some concerns
Hemmati, 2024 [39]	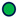	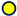	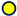	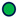	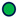	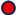	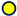	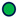	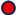	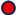	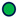	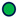	63	Some concerns
Pourhajibagher, 2022 [40]	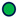	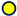	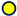	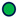	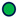	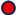	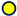	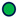	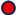	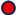	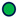	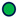	63	Some concerns
Ghorbanzadeh, [41]	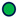	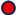	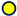	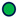	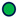	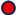	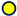	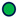	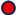	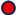	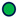	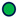	58	Some concerns
Balhaddad, 2021 [42]	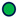	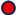	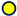	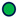	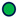	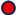	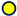	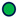	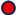	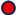	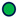	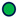	58	Some concerns
Ghanemi, 2023 [32]	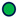	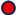	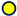	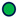	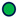	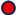	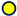	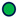	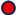	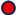	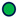	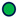	58	Some concerns
Comeau, 2022 [43]	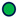	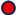	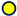	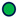	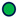	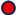	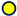	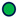	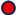	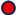	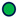	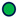	58	Some concerns
Binhasan, 2023 [44]	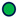	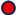	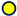	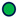	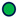	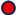	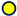	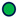	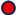	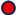	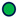	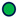	58	Some concerns
Xu, 2022 [45]	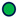	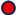	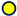	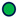	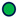	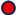	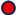	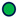	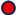	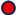	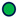	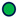	54	Some concerns
Guo, 2023 [46]	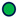	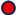	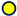	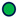	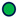	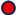	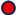	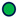	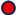	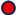	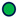	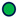	54	Some concerns
Lu, 2023 [47]	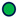	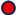	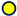	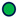	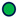	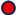	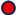	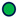	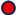	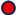	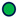	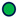	54	Some concerns
Panda, 2024 [48]	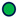	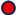	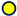	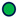	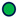	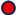	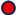	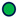	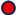	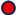	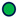	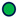	54	Some concerns
Wang, 2023 [28]	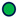	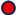	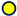	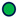	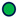	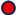	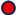	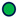	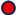	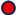	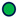	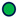	54	Some concerns
Pourhajibagher, 2022 [49]	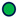	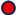	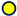	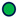	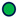	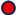	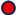	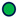	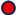	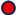	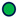	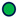	54	Some concerns
Afrasiabi, 2020 [50]	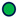	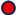	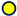	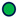	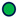	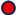	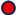	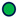	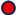	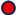	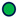	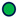	54	Some concerns
Lu, 2021 [51]	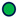	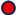	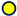	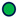	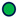	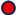	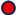	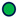	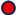	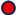	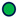	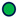	54	Some concerns
Silvestre, 2023 [52]	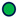	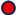	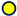	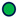	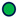	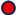	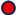	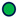	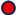	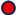	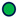	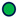	54	Some concerns
Hosseinpour-Nader, 2023 [53]	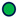	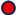	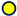	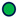	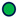	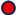	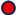	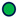	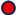	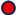	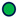	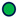	54	Some concerns
Wang, 2023 [54]	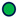	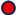	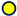	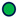	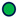	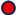	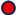	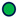	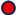	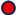	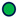	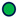	54	Some concerns
Hu, 2022 [55]	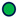	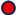	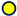	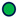	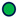	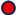	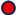	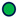	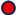	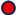	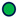	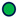	54	Some concerns
Ahrari, 2024 [56]	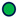	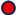	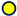	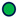	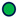	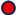	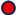	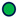	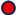	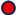	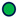	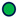	54	Some concerns
Pourhajibagher, 2019 [29]	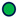	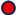	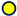	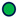	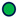	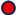	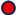	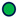	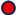	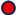	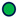	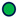	54	Some concerns
Darvish, 2024 [57]	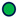	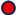	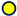	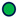	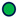	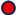	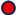	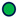	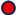	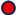	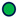	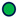	54	Some concerns
Pourhajibagher, 2021 [58]	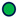	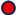	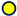	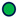	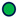	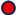	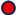	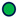	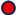	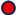	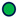	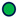	54	Some concerns
Ahrari, 2023 [59]	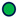	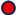	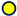	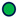	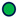	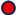	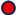	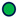	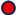	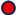	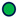	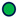	54	Some concerns
Cai, 2014 [60]	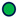	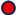	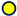	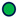	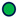	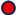	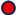	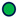	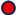	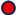	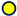	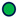	50	Some concerns
Hu, 2022 [61]	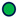	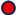	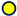	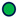	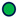	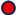	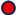	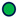	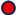	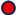	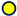	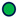	50	Some concerns
Ahmadi, 2020 [62]	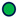	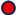	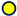	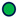	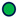	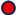	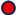	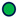	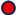	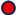	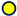	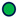	50	Some concerns

* Studies were scored as low risk (2 points, marked as 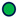), some concerns (1 point, marked as 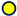), or high risk (0 points, marked as 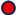). Low risk of bias: >70% score; some concerns: between 50% and 70% scores.

**Table 3:** Risk of bias of the included animal studies *.

First Author, Year [Ref.]	Items of Assessment										Score (%)	Risk of Bias
	Allocation Sequence	Baseline Similarity	Allocation Concealment	Random Housing of Animals	Caregiver/Investigator Blinding	Random Outcome Assessment	Outcome Assessor Blinding	Incomplete Data Addressed	Free of Selective Reporting	Free of Other Bias		
Zhang, 2024 [63]	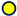	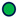	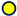	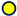	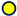	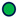	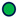	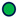	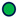	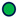	80	Low
Yu, 2022 [64]	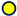	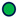	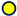	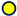	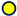	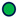	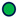	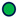	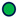	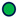	80	Low
Shi, 2025 [65]	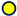	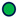	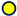	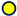	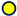	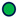	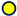	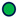	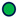	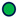	75	Low
Li, 2022 [66]	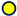	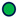	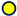	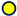	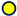	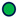	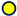	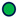	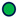	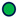	75	Low
Hajfathalian, 2023 [30]	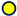	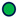	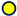	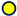	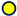	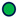	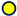	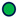	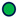	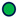	75	Low
Li, 2024 [67]	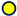	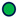	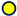	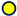	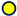	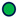	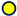	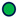	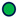	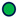	75	Low
Ran, 2024 [31]	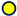	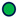	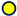	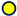	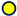	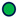	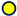	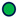	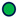	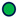	75	Low
Liu, 2022 [68]	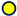	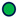	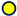	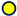	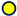	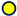	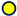	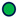	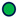	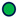	70	Low

* Studies were scored as low risk (2 points, marked as 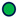) or some concerns (1 point, marked as 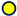). Low risk of bias: >70% score.

**Table 4:** Risk of bias of the included human study *.

First Author, Year [Ref.]	Items of Assessment					Score (%)	Risk of Bias
	Randomization Process Bias	Intervention Deviation Bias	Missing Outcome Data	Outcome Measurement Bias	Reporting Selection Bias		
Hosseinpour-Nader, 2022 [69]	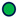	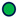	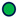	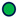	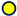	90	Low

* Studies were scored as low risk (2 points, marked as 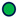) or some concerns (1 point, marked as 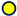). Low risk of bias: >70% score.

**Table 5:** Studies of nanomaterials used in photodynamic therapy against cariogenic microbes.

Nanomaterial [Ref.]	Microbes	Design (s)	Light Type, Wavelength	Initial Power Density	Exposure Time	Potential Use
Natural Photodynamic Nanomaterials						
Curcumin nanomicelle [59]	*L. casei*	In vitro	Broad spectrum, 450 nm	1200 mW/cm^2^	2 min	-
Curcumin nanomicelle [69]	*S. mutans*	Human	Single wavelength, 450 nm	80 mW/cm^2^	3 min	Oral capsule
Curcumin nanoparticles [52]	*S. mutans*	In vitro	Single wavelength, 460 nm	21 mW/cm^2^	12 min	Buccal film
Curcumin nanoparticles [56]	*S. mutans*	In vitro	Broad spectrum, 450 nm	1200 mW/cm^2^	2 min	Topical agent
Curcumin nanomicelle [38]	*S. mutans*, *L. acidophilus*	In vitro	Single wavelength, 450 nm	1000 mW/cm^2^	1 min	Topical agent
Curcumin nanoparticles [29]	*S. mutans*, *S. sobrinus*, *L. acidophilus*	In vitro	Broad spectrum, 435 nm	1400 mW/cm^2^	5 min	Adhesive
Curcumin nanoparticles [62]	*S. mutans*	In vitro	Broad spectrum, 405 nm	150 mW/cm^2^	-	Adhesive
Curcumin nanoparticles [58]	*S. mutans*	In vitro	Broad spectrum, 435 nm	1400 mW/cm^2^	5 min	Pulp capping agent
Emodin nanoparticles [40]	*S. mutans*	In vitro	Single wavelength, 405 nm	150 mW/cm^2^	5 min	Topical agent
Quercetin nanoparticles [49]	*S. mutans*	In vitro	Broad spectrum, 405 nm	150 mW/cm^2^	1 min	-
Quercetin nanoparticles [53]	*S. mutans*	In vitro	Broad spectrum, 450 nm	150 mW/cm^2^	5 min	Topical agent
Synthetic Photodynamic Nanomaterials						
Toluidine blue ortho nanoparticles [50]	*S. mutans*	In vitro	Single wavelength, 635 nm	220 mW/cm^2^	-	-
Toluidine blue ortho nanoparticles [48]	*S. mutans*	In vitro	Single wavelength, 650 nm	100 mW/cm^2^	2 min	-
Toluidine blue ortho nanoparticles [42]	*Saliva microbes*	In vitro	Broad spectrum, 668 nm	600 mW/cm^2^	5 min	Topical agent
Methylene blue nanoparticles [37]	*S. mutans*	In vitro	Single wavelength, 638 nm	1500 mW/cm^2^	30 s	Adhesive
Methylene blue nanoparticles [44]	*L. acidophilus*	In vitro	Single wavelength, 660 nm	40 mW/cm^2^	1 min	Topical agent
Chlorin e6 nanoparticles [68]	*S. mutans*, *S. sobrinus*, *S. sanguinis*	In vitro, animal	Single wavelength, 660 nm	500 mW/cm^2^	5 min	Topical agent
Chlorin e6 nanoparticles [54]	*S. mutans*	In vitro	Single wavelength, 650 nm	120 mW/cm^2^	-	-
Chlorin e6 nanoparticles [46]	*S. mutans*	In vitro	Single wavelength, 660 nm	100 mW/cm^2^	30 min	Topical agent
Chlorin e6 nanoparticles [65]	*S. mutans*, *S. sanguis*, *C. albicans*	In vitro, animal	Single wavelength, 665 nm	214 mW/cm^2^	5 min	Gargle
Zinc oxide nanoparticles [39]	*S. mutans*	In vitro	Broad spectrum, 450 nm	1400 mW/cm^2^	1 min	Adhesive
Zinc oxide nanoparticles [43]	*S. mutans*	In vitro	Broad spectrum, 450 nm	23 mW/cm^2^	1 min	Restorative filler
Titanium dioxide nanoparticles [28]	*S. mutans*	In vitro	Broad spectrum, 450 nm	150 mW/cm^2^	5 min	Topical agent
Titanium dioxide nanoparticles [60]	*S. mutans*	In vitro	Broad spectrum, 371 nm	12 mW/cm^2^	25 h	Adhesive
Bismuth oxychloride nanoparticles [66]	*S. aureus*, *S. mutans*, *E. coli*	In vitro, animal	-, Green light	-	5 min	Topical agent

**Table 6:** Studies of copper salt materials against cariogenic microorganism.

Nanomaterial [Ref.]	Microbes	Design (s)	Light Type, Wavelength	Initial Power Density	Exposure Time	Potential Use
Metal-Based Photothermal Nanomaterials						
Gold nanoparticles [57]	*S. mutans*	In vitro	Single wavelength, 660 nm	100 mW/cm^2^	30 s	Restorative filler
Gold nanoparticles [30]	*S. mutans*, *S. aureus*	In vitro, animal	Single wavelength, 808 nm	500 mW/cm^2^	5 min	Topical agent
Bismuth nanoparticles [55]	*S. mutans*	In vitro	Single wavelength, -	12 mW/cm^2^	24 h	Restorative filler
Iron (III) ferrocyanide nanoparticles [67]	*S. mutans*, *S. sobrinus*, *S. sanguinis*	In vitro, animal	Single wavelength, 808 nm	400 mW/cm^2^	3 min	Topical agent
Titanium aluminium carbide nanoparticles [61]	*S. mutans*	In vitro	Broad spectrum, Natural light	-	5 min	Restorative filler
Carbon-Based Photothermal Nanomaterials						
Graphene oxide nanosheets [51]	*S. mutans*	In vitro	Single wavelength, 808 nm	880 mW/cm^2^	5 min	-
Polydopamine nanoparticles [45]	*S. mutans*	In vitro	Single wavelength, 808 nm	750 mW/cm^2^	10 min	-
Polydopamine nanoparticles [47]	*S. mutans*	In vitro	Single wavelength, 808 nm	1500 mW/cm^2^	10 min	Topical agent
Phosphorus-Based Photothermal Nanomaterials						
Black phosphorus nanosheets [31]	*S. mutans*, *S. sanguinis*	In vitro, animal	Single wavelength, 808 nm	1000 mW/cm^2^	5 min	Topical agent

**Table 7:** Studies of nanomaterials in combined photothermal and photodynamic therapy against cariogenic microbes.

Nanomaterial [Ref.]	Microbes	Design (s)	Light Type, Wavelength	Initial Power Density	Exposure Time	Potential Use
Metal-Based Dual-Modal Nanomaterials						
Zinc phthalocyanine tetrasulfonate nanoparticles [63]	*S. mutans*	In vitro, animal	Single wavelength, 660 nm	1000 mW/cm^2^	5 min	Topical agent
Carbon-Based Dual-Modal Nanomaterials						
Graphene oxide nanoparticles [41]	*S. mutans*	In vitro	Single wavelength, 980 nm	500 mW/cm^2^	5 min	Adhesive
Reduced graphene oxide nanosheets [32]	*S. mutans*	In vitro	Single wavelength, 980 nm	1635 mW/cm^2^	2 min	Coating
Poly(2-(5,5-dimethyl-1,3-dioxan-2-yloxy)ethyl acrylate) [64]	*S. mutans*	In vitro, animal	Single wavelength, 808 nm	1500 mW/cm^2^	5 min	Topical agent

## Data Availability

No new data were created or analyzed in this study. Data sharing is not applicable to this article.

## Supplementary Materials

The following supporting information can be downloaded at: https://www.mdpi.com/article/10.3390/jfb16090308/s1, Preferred Reporting Items for Systematic reviews and Meta-Analyses extension for Scoping Reviews (PRISMA-ScR) Checklist.

## References

[1] Pitts N.B., Zero D.T., Marsh P.D., Ekstrand K., Weintraub J.A., Ramos-Gomez F., Tagami J., Twetman S., Tsakos G., Ismail A. (2017). Dental caries. Nat. Rev. Dis. Primers.

[2] GBD 2017 Disease and Injury Incidence and Prevalence Collaborators (2018). Global, regional, and national incidence, prevalence, and years lived with disability for 354 diseases and injuries for 195 countries and territories, 1990–2017: A systematic analysis for the Global Burden of Disease Study 2017. Lancet.

[3] Selwitz R.H., Ismail A.I., Pitts N.B. (2007). Dental caries. Lancet.

[4] Xu V.W., Yin I.X., Niu J.Y., Yu O.Y., Nizami M.Z.I., Chu C.H. (2024). Developing a novel antibacterial copper tetraamine fluoride. J. Dent..

[5] Xu V.W., Nizami M.Z.I., Yin I.X., Niu J.Y., Yu O.Y., Chu C.H. (2023). Copper Materials for Caries Management: A Scoping Review. J. Funct. Biomater..

[6] Ahmadian E., Shahi S., Yazdani J., Maleki Dizaj S., Sharifi S. (2018). Local treatment of the dental caries using nanomaterials. Biomed. Pharmacother..

[7] Schwendicke F., Paris S., Tu Y.K. (2015). Effects of using different criteria for caries removal: A systematic review and network meta-analysis. J. Dent..

[8] Whelton H.P., Spencer A.J., Do L.G., Rugg-Gunn A.J. (2019). Fluoride Revolution and Dental Caries: Evolution of Policies for Global Use. J. Dent. Res..

[9] Qiu W., Zhou Y., Li Z., Huang T., Xiao Y., Cheng L., Peng X., Zhang L., Ren B. (2020). Application of Antibiotics/Antimicrobial Agents on Dental Caries. Biomed. Res. Int..

[10] Kalka K., Merk H., Mukhtar H. (2000). Photodynamic therapy in dermatology. J. Am. Acad. Dermatol..

[11] Dolmans D.E., Fukumura D., Jain R.K. (2003). Photodynamic therapy for cancer. Nat. Rev. Cancer.

[12] Sun J., Song L., Fan Y., Tian L., Luan S., Niu S., Ren L., Ming W., Zhao J. (2019). Synergistic Photodynamic and Photothermal Antibacterial Nanocomposite Membrane Triggered by Single NIR Light Source. ACS Appl. Mater. Interfaces.

[13] Warrier A., Mazumder N., Prabhu S., Satyamoorthy K., Murali T.S. (2021). Photodynamic therapy to control microbial biofilms. Photodiagn. Photodyn. Ther..

[14] Overchuk M., Weersink R.A., Wilson B.C., Zheng G. (2023). Photodynamic and Photothermal Therapies: Synergy Opportunities for Nanomedicine. ACS Nano.

[15] Ren Y., Yan Y., Qi H. (2022). Photothermal conversion and transfer in photothermal therapy: From macroscale to nanoscale. Adv. Colloid. Interface Sci..

[16] Qi M., Chi M., Sun X., Xie X., Weir M.D., Oates T.W., Zhou Y., Wang L., Bai Y., Xu H.H. (2019). Novel nanomaterial-based antibacterial photodynamic therapies to combat oral bacterial biofilms and infectious diseases. Int. J. Nanomed..

[17] Konopka K., Goslinski T. (2007). Photodynamic therapy in dentistry. J. Dent. Res..

[18] da Mota A.C., Leal C.R., Olivan S., Leal Gonçalves M.L., de Oliveira V.A., Pinto M.M., Bussadori S.K. (2016). Case Report of Photodynamic Therapy in the Treatment of Dental Caries on Primary Teeth. J. Lasers Med. Sci..

[19] Li Y., Jiao J., Qi Y., Yu W., Yang S., Zhang J., Zhao J. (2021). Curcumin: A review of experimental studies and mechanisms related to periodontitis treatment. J. Periodontal Res..

[20] Kumari S., Sharma N., Sahi S.V. (2021). Advances in Cancer Therapeutics: Conventional Thermal Therapy to Nanotechnology-Based Photothermal Therapy. Pharmaceutics.

[21] Xu V.W., Nizami M.Z.I., Yin I.X., Lung C.Y.K., Yu O.Y., Chu C.H. (2022). Caries Management with Non-Metallic Nanomaterials: A Systematic Review. Int. J. Nanomed..

[22] Raza F., Zafar H., Zhang S., Kamal Z., Su J., Yuan W.E., Mingfeng Q. (2021). Recent Advances in Cell Membrane-Derived Biomimetic Nanotechnology for Cancer Immunotherapy. Adv. Heal. Mater..

[23] Awad M., Thomas N., Barnes T.J., Prestidge C.A. (2022). Nanomaterials enabling clinical translation of antimicrobial photodynamic therapy. J. Control Release.

[24] Kesharwani P., Ma R., Sang L., Fatima M., Sheikh A., Abourehab M.A.S., Gupta N., Chen Z.S., Zhou Y. (2023). Gold nanoparticles and gold nanorods in the landscape of cancer therapy. Mol. Cancer.

[25] Dilenko H., Bartoň Tománková K., Válková L., Hošíková B., Kolaříková M., Malina L., Bajgar R., Kolářová H. (2024). Graphene-Based Photodynamic Therapy and Overcoming Cancer Resistance Mechanisms: A Comprehensive Review. Int. J. Nanomed..

[26] Lucky S.S., Soo K.C., Zhang Y. (2015). Nanoparticles in photodynamic therapy. Chem. Rev..

[27] Silvestre A.L.P., Di Filippo L.D., Besegato J.F., de Annunzio S.R., Almeida Furquim de Camargo B., de Melo P.B.G., Rastelli A.N.S., Fontana C.R., Chorilli M. (2021). Current applications of drug delivery nanosystems associated with antimicrobial photodynamic therapy for oral infections. Int. J. Pharm..

[28] Wang R., Jia C., Zheng N., Liu S., Qi Z., Wang R., Zhang L., Niu Y., Pan S. (2023). Effects of photodynamic therapy on Streptococcus mutans and enamel remineralization of multifunctional TiO(2)-HAP composite nanomaterials. Photodiagn. Photodyn. Ther..

[29] Pourhajibagher M., Salehi Vaziri A., Takzaree N., Ghorbanzadeh R. (2019). Physico-mechanical and antimicrobial properties of an orthodontic adhesive containing cationic curcumin doped zinc oxide nanoparticles subjected to photodynamic therapy. Photodiagn. Photodyn. Ther..

[30] Hajfathalian M., de Vries C.R., Hsu J.C., Amirshaghaghi A., Dong Y.C., Ren Z., Liu Y., Huang Y., Li Y., Knight S.A. (2023). Theranostic gold-in-gold cage nanoparticles enable photothermal ablation and photoacoustic imaging in biofilm-associated infection models. J. Clin. Investig..

[31] Ran Y., Shi J., Ding Y., Li L., Lu D., Zeng Y., Qiu D., Yu J., Cai X., Pan Y. (2024). Black Phosphorus Nanosheets-Loaded Mussel-Inspired Hydrogel with Wet Adhesion, Photothermal Antimicrobial, and In Situ Remineralization Capabilities for Caries Prevention. Adv. Sci..

[32] Ghanemi M., Salehi-Vaziri A., Pourhajibagher M., Bahador A. (2023). Physico-mechanical and antimicrobial properties of an elastomeric ligature coated with reduced nanographene oxide-nano curcumin subjected to dual-modal photodynamic and photothermal inactivation against Streptococcus mutans biofilms. Photodiagn. Photodyn. Ther..

[33] Tricco A.C., Lillie E., Zarin W., O′Brien K.K., Colquhoun H., Levac D., Moher D., Peters M.D., Horsley T., Weeks L. (2018). PRISMA extension for scoping reviews (PRISMA-ScR): Checklist and explanation. Ann. Intern. Med..

[34] Sheth V.H., Shah N.P., Jain R., Bhanushali N., Bhatnagar V. (2024). Development and validation of a risk-of-bias tool for assessing in vitro studies conducted in dentistry: The QUIN. J. Prosthet. Dent..

[35] Hooijmans C.R., Rovers M.M., de Vries R.B.M., Leenaars M., Ritskes-Hoitinga M., Langendam M.W. (2014). SYRCLE’s risk of bias tool for animal studies. BMC Med. Res. Methodol..

[36] Sterne J.A.C., Savović J., Page M.J., Elbers R.G., Blencowe N.S., Boutron I., Cates C.J., Cheng H.Y., Corbett M.S., Eldridge S.M. (2019). RoB 2: A revised tool for assessing risk of bias in randomised trials. Bmj.

[37] Alanazi A.M., Khan N.A., Khan A.A., Bhutto K., Askary S.H., Askary G., Abrar E., Mahmood S.J., Qureshi A. (2024). Titanium oxide and chitosan nanoparticles loaded in methylene blue activated by photodynamic therapy on caries affected dentin disinfection, bond strength, and smear layer removal efficacy. Photodiagn. Photodyn. Ther..

[38] Afrasiabi S., Al Gburi A.Q.K., Ranjbar Omrani L., Chiniforush N., Moradi Z. (2024). Evaluation of riboflavin, nanocurcumin, and hydrogen peroxide under light conditions: Reduction of mature dental biofilms and enamel mineral loss. Photodiagn. Photodyn. Ther..

[39] Hemmati Y.B., Bahrami R., Pourhajibagher M. (2024). Assessing the physico-mechanical, anti-bacterial, and anti-demineralization properties of orthodontic resin composite containing different concentrations of photoactivated zinc oxide nanoparticles on Streptococcus mutans biofilm around ceramic and metal orthodontic brackets: An ex vivo study. Int. Orthod..

[40] Pourhajibagher M., Keshavarz Valian N., Bahador A. (2022). Theranostic nanoplatforms of emodin-chitosan with blue laser light on enhancing the anti-biofilm activity of photodynamic therapy against Streptococcus mutans biofilms on the enamel surface. BMC Microbiol..

[41] Ghorbanzadeh R., Hosseinpour Nader A., Salehi-Vaziri A. (2021). The effects of bimodal action of photodynamic and photothermal therapy on antimicrobial and shear bond strength properties of orthodontic composite containing nano-graphene oxide. Photodiagn. Photodyn. Ther..

[42] Balhaddad A.A., Xia Y., Lan Y., Mokeem L., Ibrahim M.S., Weir M.D., Xu H.H.K., Melo M.A.S. (2021). Magnetic-Responsive Photosensitizer Nanoplatform for Optimized Inactivation of Dental Caries-Related Biofilms: Technology Development and Proof of Principle. ACS Nano.

[43] Comeau P., Burgess J., Malekafzali N., Leite M.L., Lee A., Manso A. (2022). Exploring the Physicochemical, Mechanical, and Photocatalytic Antibacterial Properties of a Methacrylate-Based Dental Material Loaded with ZnO Nanoparticles. Materials.

[44] Binhasan M., Alsunbul H., Aljanakh M., Abduljabbar T., Vohra F. (2023). Dentin disinfection and adhesive bond strength using modified photoactivated carbon nanoparticles. Photodiagn. Photodyn. Ther..

[45] Xu X.Y., Fan M.L., Yu Z.H., Zhao Y., Zhang H.B., Wang J., Wu M.Z., Sun F., Xu X.N., Ding C.M. (2022). A removable photothermal antibacterial “warm paste” target for cariogenic bacteria. Chem. Eng. J..

[46] Guo W., Li Y., Wang S., Wang Y., Li C., Jin Y., Li Y., Chen X., Miao W. (2023). Photodynamic nano hydroxyapatite with biofilm penetration capability for dental plaque eradication and prevention of demineralization. Colloids Surf. B Biointerfaces.

[47] Lu X., Qu Y., Zhu T., Qu X., Zhang Z., Yu Y., Hao Y. (2023). Applications of photothermally mediated nanohybrids for white spot lesions in orthodontics. Colloids Surf. B Biointerfaces.

[48] Panda S., Rout L., Mohanty N., Satpathy A., Sankar Satapathy B., Rath S., Gopinath D. (2024). Exploring the photosensitizing potential of Nanoliposome Loaded Improved Toluidine Blue O (NLITBO) Against Streptococcus mutans: An in-vitro feasibility study. PLoS ONE.

[49] Pourhajibagher M., Alaeddini M., Etemad-Moghadam S., Rahimi Esboei B., Bahrami R., Miri Mousavi R.S., Bahador A. (2022). Quorum quenching of Streptococcus mutans via the nano-quercetin-based antimicrobial photodynamic therapy as a potential target for cariogenic biofilm. BMC Microbiol..

[50] Afrasiabi S., Pourhajibagher M., Chiniforush N., Bahador A. (2020). Propolis nanoparticle enhances the potency of antimicrobial photodynamic therapy against Streptococcus mutans in a synergistic manner. Sci. Rep..

[51] Lu B.Y., Zhu G.Y., Yu C.H., Chen G.Y., Zhang C.L., Zeng X., Chen Q.M., Peng Q. (2021). Functionalized graphene oxide nanosheets with unique three-in-one properties for efficient and tunable antibacterial applications. Nano Res..

[52] Silvestre A.L.P., Dos Santos A.M., de Oliveira A.B., Ferrisse T.M., Brighenti F.L., Meneguin A.B., Chorilli M. (2023). Evaluation of photodynamic therapy on nanoparticles and films loaded-nanoparticles based on chitosan/alginate for curcumin delivery in oral biofilms. Int. J. Biol. Macromol..

[53] Hosseinpour-Nader A., Karimi N., Ghafari H.A. (2023). Ex-vivo effects of propolis quantum dots-nisin-nanoquercetin-mediated photodynamic therapy on Streptococcus mutans biofilms and white spot lesions. Photodiagn. Photodyn. Ther..

[54] Wang R., Pan Q., Li F., Guo J., Huo Y., Xu C., Xiong M., Cheng Z., Liu M., Lin J. (2023). Oxygen-carrying acid-responsive Cu/ZIF-8 for photodynamic antibacterial therapy against cariogenic Streptococcus mutans infection. Dalton Trans..

[55] Hu Y., Xu Z., Hu Y., Hu L., Zi Y., Wang M., Feng X., Huang W. (2022). Bismuth Quantum Dot (Bi QD)/Polydimethylsiloxane (PDMS) Nanocomposites with Self-Cleaning and Antibacterial Activity for Dental Applications. Nanomaterials.

[56] Ahrari F., Nazifi M., Mazhari F., Ghazvini K., Menbari S., Fekrazad R., Babaei K., Banihashemrad A. (2024). Photoinactivation Effects of Curcumin, Nano-curcumin, and Erythrosine on Planktonic and Biofilm Cultures of Streptococcus mutans. J. Lasers Med. Sci..

[57] Darvish S., Budala D.G., Goriuc A. (2024). Antibacterial Properties of an Experimental Dental Resin Loaded with Gold Nanoshells for Photothermal Therapy Applications. J. Funct. Biomater..

[58] Pourhajibagher M., Ranjbar Omrani L., Noroozian M., Ghorbanzadeh Z., Bahador A. (2021). In vitro antibacterial activity and durability of a nano-curcumin-containing pulp capping agent combined with antimicrobial photodynamic therapy. Photodiagn. Photodyn. Ther..

[59] Ahrari F., Mazhari F., Ghazvini K., Fekrazad R., Menbari S., Nazifi M. (2023). Antimicrobial photodynamic therapy against Lactobacillus casei using curcumin, nano-curcumin, or erythrosine and a dental LED curing device. Lasers Med. Sci..

[60] Cai Y., Strømme M., Melhus A., Engqvist H., Welch K. (2014). Photocatalytic inactivation of biofilms on bioactive dental adhesives. J. Biomed. Mater. Res. B Appl. Biomater..

[61] Hu Y., Xu Z., Pu J., Hu L., Zi Y., Wang M., Feng X., Huang W. (2022). 2D MXene Ti(3)C(2)T (x) nanosheets in the development of a mechanically enhanced and efficient antibacterial dental resin composite. Front. Chem..

[62] Ahmadi H., Haddadi-Asl V., Ghafari H.A., Ghorbanzadeh R., Mazlum Y., Bahador A. (2020). Shear bond strength, adhesive remnant index, and anti-biofilm effects of a photoexcited modified orthodontic adhesive containing curcumin doped poly lactic-co-glycolic acid nanoparticles: An ex-vivo biofilm model of S. mutans on the enamel slab bonded brackets. Photodiagn. Photodyn. Ther..

[63] Zhang Y., Jiang Z.T., Wang Y., Wang H.Y., Hong S., Li W., Guo D.S., Zhang X. (2024). A Supramolecular Nanoformulation with Adaptive Photothermal/Photodynamic Transformation for Preventing Dental Caries. ACS Nano.

[64] Yu Y.J., Zhang Y.F., Cheng Y.J., Wang Y.X., Chen Z.Y., Sun H.N., Wei X.S., Ma Z., Li J., Bai Y.Y. (2022). NIR-activated nanosystems with self-modulated bacteria targeting for enhanced biofilm eradication and caries prevention. Bioact. Mater..

[65] Shi J., Qi X., Ran Y., Zhou Q., Ding Y., Li L., Zeng Y., Qiu D., Cai Z., Cai X. (2025). Saliva-acquired pellicle inspired multifunctional gargle with wet adhesion, photodynamic antimicrobial, and In situ remineralization properties for dental caries prevention. Bioact. Mater..

[66] Li Q., Liu J., Xu Y., Liu H., Zhang J., Wang Y., Sun Y., Zhao M., Liao L., Wang X. (2022). Fast Cross-Linked Hydrogel as a Green Light-Activated Photocatalyst for Localized Biofilm Disruption and Brush-Free Tooth Whitening. ACS Appl. Mater. Interfaces.

[67] Li S., Li Q., Zhang H., Li F., Hu J., Qian J., Wang Y., Zhang J., Wu Z. (2024). Dental Caries Management with Antibacterial Silver-Doped Prussian Blue Hydrogel by the Combined Effects of Photothermal Response and Ion Discharge. ACS Appl. Mater. Interfaces.

[68] Liu D., Ma X., Ji Y., Chen R., Zhou S., Yao H., Zhang Z., Ye M., Xu Z., Du M. (2022). Bioresponsive nanotherapy for preventing dental caries by inhibiting multispecies cariogenic biofilms. Bioact. Mater..

[69] Hosseinpour-Nader A., Karimi N., Ghafari H.A., Ghorbanzadeh R. (2022). Effect of nanomicelle curcumin-based photodynamic therapy on the dynamics of white spot lesions and virulence of Streptococcus mutans in patients undergoing fixed orthodontic treatment: A randomized double-blind clinical trial. Photodiagn. Photodyn. Ther..

[70] Agostinis P., Berg K., Cengel K.A., Foster T.H., Girotti A.W., Gollnick S.O., Hahn S.M., Hamblin M.R., Juzeniene A., Kessel D. (2011). Photodynamic therapy of cancer: An update. CA. Cancer J. Clin..

[71] Kwiatkowski S., Knap B., Przystupski D., Saczko J., Kędzierska E., Knap-Czop K., Kotlińska J., Michel O., Kotowski K., Kulbacka J. (2018). Photodynamic therapy—Mechanisms, photosensitizers and combinations. Biomed. Pharmacother..

[72] Kolarikova M., Hosikova B., Dilenko H., Barton-Tomankova K., Valkova L., Bajgar R., Malina L., Kolarova H. (2023). Photodynamic therapy: Innovative approaches for antibacterial and anticancer treatments. Med. Res. Rev..

[73] Badran Z., Rahman B., De Bonfils P., Nun P., Coeffard V., Verron E. (2023). Antibacterial nanophotosensitizers in photodynamic therapy: An update. Drug Discov. Today.

[74] Balhaddad A.A., Garcia I.M., Ibrahim M.S., Rolim J., Gomes E.A.B., Martinho F.C., Collares F.M., Xu H., Melo M.A.S. (2020). Prospects on Nano-Based Platforms for Antimicrobial Photodynamic Therapy Against Oral Biofilms. Photobiomodul Photomed. Laser Surg..

[75] Yan R., Liu J., Dong Z., Peng Q. (2023). Nanomaterials-mediated photodynamic therapy and its applications in treating oral diseases. Biomater. Adv..

[76] Chen J., Fan T., Xie Z., Zeng Q., Xue P., Zheng T., Chen Y., Luo X., Zhang H. (2020). Advances in nanomaterials for photodynamic therapy applications: Status and challenges. Biomaterials.

[77] Abrahamse H., Hamblin M.R. (2016). New photosensitizers for photodynamic therapy. Biochem. J..

[78] Baghdan E., Duse L., Schüer J.J., Pinnapireddy S.R., Pourasghar M., Schäfer J., Schneider M., Bakowsky U. (2019). Development of inhalable curcumin loaded Nano-in-Microparticles for bronchoscopic photodynamic therapy. Eur. J. Pharm. Sci..

[79] Santezi C., Reina B.D., Dovigo L.N. (2018). Curcumin-mediated Photodynamic Therapy for the treatment of oral infections-A review. Photodiagn. Photodyn. Ther..

[80] Etemadi A., Hamidain M., Parker S., Chiniforush N. (2021). Blue Light Photodynamic Therapy With Curcumin and Riboflavin in the Management of Periodontitis: A Systematic Review. J. Lasers Med. Sci..

[81] Ensafi F., Fazlyab M., Chiniforush N., Akhavan H. (2022). Comparative effects of SWEEPS technique and antimicrobial photodynamic therapy by using curcumin and nano-curcumin on Enterococcus faecalis biofilm in root canal treatment. Photodiagn. Photodyn. Ther..

[82] Duse L., Agel M.R., Pinnapireddy S.R., Schäfer J., Selo M.A., Ehrhardt C., Bakowsky U. (2019). Photodynamic Therapy of Ovarian Carcinoma Cells with Curcumin-Loaded Biodegradable Polymeric Nanoparticles. Pharmaceutics.

[83] Yaghobee S., Pourhajibagher M., Bahrami R., Isaabadi M. (2024). Nano-emodin mediated photodynamic therapy for wound healing of donor site after free gingival graft: A parallel clinical trial. Photodiagn. Photodyn. Ther..

[84] Monem Moharrer S., Pourhajibagher M., Azizi A., Alaee A. (2025). Anti-virulence effect of photoactivated nano-quercetin by diode laser on Aggregatibacter actinomycetemcomitans. AMB Express.

[85] Najm M., Pourhajibagher M., Badirzadeh A., Razmjou E., Alipour M., Khoshmirsafa M., Bahador A., Hadighi R. (2021). Photodynamic Therapy Using Toluidine Blue O (TBO) Dye as a Photosensitizer against Leishmania major. Iran J. Public Health.

[86] Rout B., Liu C.H., Wu W.C. (2018). Increased anti-biofilm efficacy of toluidine blue on Staphylococcus species after nano-encapsulation. Photodiagn. Photodyn. Ther..

[87] Lim D.J. (2021). Methylene Blue-Based Nano and Microparticles: Fabrication and Applications in Photodynamic Therapy. Polym..

[88] Zada L., Anwar S., Imtiaz S., Saleem M., Shah A.A. (2024). In vitro study: Methylene blue-based antibacterial photodynamic inactivation of Pseudomonas aeruginosa. Appl. Microbiol. Biotechnol..

[89] Ding Y.F., Li S., Liang L., Huang Q., Yuwen L., Yang W., Wang R., Wang L.H. (2018). Highly Biocompatible Chlorin e6-Loaded Chitosan Nanoparticles for Improved Photodynamic Cancer Therapy. ACS Appl. Mater. Interfaces.

[90] Hu X., Tian H., Jiang W., Song A., Li Z., Luan Y. (2018). Rational Design of IR820- and Ce6-Based Versatile Micelle for Single NIR Laser-Induced Imaging and Dual-Modal Phototherapy. Small.

[91] Ancona A., Dumontel B., Garino N., Demarco B., Chatzitheodoridou D., Fazzini W., Engelke H., Cauda V. (2018). Lipid-Coated Zinc Oxide Nanoparticles as Innovative ROS-Generators for Photodynamic Therapy in Cancer Cells. Nanomaterials.

[92] Ziental D., Czarczynska-Goslinska B., Mlynarczyk D.T., Glowacka-Sobotta A., Stanisz B., Goslinski T., Sobotta L. (2020). Titanium Dioxide Nanoparticles: Prospects and Applications in Medicine. Nanomaterials.

[93] Liu Y., Wang Z., Liu X., Chen H., Huang Y., Li A., Pu Y., Guo L. (2025). Study on Mechanical Properties, Optical Properties, Cytotoxicity of TiO(2)-HAP Nanoparticles-Modified PMMA and Photodynamically Assisted Antibacterial Activity Against Candida Albicans in Vitro. Int. J. Nanomed..

[94] Lucky S.S., Idris N.M., Huang K., Kim J., Li Z., Thong P.S., Xu R., Soo K.C., Zhang Y. (2016). In vivo Biocompatibility, Biodistribution and Therapeutic Efficiency of Titania Coated Upconversion Nanoparticles for Photodynamic Therapy of Solid Oral Cancers. Theranostics.

[95] Tian Y., Li Y., Liu J., Lin Y., Jiao J., Chen B., Wang W., Wu S., Li C. (2022). Photothermal therapy with regulated Nrf2/NF-κB signaling pathway for treating bacteria-induced periodontitis. Bioact. Mater..

[96] Chen Y., Gao Y., Chen Y., Liu L., Mo A., Peng Q. (2020). Nanomaterials-based photothermal therapy and its potentials in antibacterial treatment. J. Control. Release.

[97] Gupta N., Malviya R. (2021). Understanding and advancement in gold nanoparticle targeted photothermal therapy of cancer. Biochim. Biophys. Acta Rev. Cancer.

[98] Dheyab M.A., Aziz A.A., Khaniabadi P.M., Jameel M.S., Oladzadabbasabadi N., Rahman A.A., Braim F.S., Mehrdel B. (2023). Gold nanoparticles-based photothermal therapy for breast cancer. Photodiagn. Photodyn. Ther..

[99] Du J., Ding H., Fu S., Li D., Yu B. (2022). Bismuth-coated 80S15C bioactive glass scaffolds for photothermal antitumor therapy and bone regeneration. Front. Bioeng. Biotechnol..

[100] Singh P., Pandit S., Mokkapati V., Garg A., Ravikumar V., Mijakovic I. (2018). Gold Nanoparticles in Diagnostics and Therapeutics for Human Cancer. Int. J. Mol. Sci..

[101] Wu S., Li A., Zhao X., Zhang C., Yu B., Zhao N., Xu F.J. (2019). Silica-Coated Gold-Silver Nanocages as Photothermal Antibacterial Agents for Combined Anti-Infective Therapy. ACS Appl. Mater. Interfaces.

[102] Nirmal G.R., Lin Z.C., Chiu T.S., Alalaiwe A., Liao C.C., Fang J.Y. (2024). Chemo-photothermal therapy of chitosan/gold nanorod clusters for antibacterial treatment against the infection of planktonic and biofilm MRSA. Int. J. Biol. Macromol..

[103] Dong L., Huang W., Chu H., Li Y., Wang Y., Zhao S., Li G., Zhang H., Li D. (2020). Passively Q-switched near-infrared lasers with bismuthene quantum dots as the saturable absorber. Opt. Laser Technol..

[104] Gomez C., Hallot G., Laurent S., Port M. (2021). Medical Applications of Metallic Bismuth Nanoparticles. Pharmaceutics.

[105] Tang K., Li X., Hu Y., Zhang X., Lu N., Fang Q., Shao J., Li S., Xiu W., Song Y. (2023). Recent advances in Prussian blue-based photothermal therapy in cancer treatment. Biomater. Sci..

[106] Li N., Wang Y., Li Y., Zhang C., Fang G. (2024). Recent Advances in Photothermal Therapy at Near-Infrared-II Based on 2D MXenes. Small.

[107] de Melo-Diogo D., Lima-Sousa R., Alves C.G., Correia I.J. (2019). Graphene family nanomaterials for application in cancer combination photothermal therapy. Biomater. Sci..

[108] Han L., Li P., Tang P., Wang X., Zhou T., Wang K., Ren F., Guo T., Lu X. (2019). Mussel-inspired cryogels for promoting wound regeneration through photobiostimulation, modulating inflammatory responses and suppressing bacterial invasion. Nanoscale.

[109] Zhao Y., Peng X., Wang D., Zhang H., Xin Q., Wu M., Xu X., Sun F., Xing Z., Wang L. (2022). Chloroplast-inspired scaffold for infected bone defect therapy: Towards stable photothermal properties and self-defensive functionality. Adv. Sci..

[110] Shao J., Ruan C., Xie H., Chu P.K., Yu X.F. (2020). Photochemical activity of black phosphorus for near-infrared light controlled in situ biomineralization. Adv. Sci..

[111] Wen F., Li P., Meng H., Yan H., Huang X., Cui H., Su W. (2022). Nitrogen-doped carbon dots/curcumin nanocomposite for combined Photodynamic/photothermal dual-mode antibacterial therapy. Photodiagn. Photodyn. Ther..

[112] An Q., Su S., Hu W., Wang Y., Liang T., Li X., Li C. (2023). Dual-wavelength responsive CuS@COF nanosheets for high-performance photothermal/photodynamic combination treatments. Nanoscale.

[113] Luo T., Nash G.T., Xu Z., Jiang X., Liu J., Lin W. (2021). Nanoscale Metal-Organic Framework Confines Zinc-Phthalocyanine Photosensitizers for Enhanced Photodynamic Therapy. J. Am. Chem. Soc..

[114] Wang Y., Li J., Li X., Shi J., Jiang Z., Zhang C.Y. (2022). Graphene-based nanomaterials for cancer therapy and anti-infections. Bioact. Mater..

[115] Sarcan E.T., Silindir-Gunay M., Ozer A.Y. (2018). Theranostic polymeric nanoparticles for NIR imaging and photodynamic therapy. Int. J. Pharm..

[116] AlRyalat S.A.S., Malkawi L.W., Momani S.M. (2019). Comparing Bibliometric Analysis Using PubMed, Scopus, and Web of Science Databases. JOVE-J. Vis. Exp..

[117] Falagas M.E., Pitsouni E.I., Malietzis G.A., Pappas G. (2008). Comparison of PubMed, Scopus, web of science, and Google scholar: Strengths and weaknesses. FASEB J..

[118] Lazarinis F., Vilares J., Tait J., Efthimiadis E.N. (2009). Current research issues and trends in non-English Web searching. Inf. Retr..

[119] Amano T., Berdejo-Espinola V., Christie A.P., Willott K., Akasaka M., Báldi A., Berthinussen A., Bertolino S., Bladon A.J., Chen M. (2021). Tapping into non-English-language science for the conservation of global biodiversity. PLoS Biol..

[120] Sreenivasalu P.K.P., Dora C.P., Swami R., Jasthi V.C., Shiroorkar P.N., Nagaraja S., Asdaq S.M.B., Anwer M.K. (2022). Nanomaterials in Dentistry: Current Applications and Future Scope. Nanomaterials.

[121] Hannig M., Hannig C. (2010). Nanomaterials in preventive dentistry. Nat. Nanotechnol..

